# iAB-RBC-283: A proteomically derived knowledge-base of erythrocyte metabolism that can be used to simulate its physiological and patho-physiological states

**DOI:** 10.1186/1752-0509-5-110

**Published:** 2011-07-12

**Authors:** Aarash Bordbar, Neema Jamshidi, Bernhard O Palsson

**Affiliations:** 1 Department of Bioengineering, University of California San Diego, 417 Powell-Focht Bioengineering Hall, 9500 Gilman Drive, La Jolla, CA, 92093-0412, USA

## Abstract

**Background:**

The development of high-throughput technologies capable of whole cell measurements of genes, proteins, and metabolites has led to the emergence of systems biology. Integrated analysis of the resulting omic data sets has proved to be hard to achieve. Metabolic network reconstructions enable complex relationships amongst molecular components to be represented formally in a biologically relevant manner while respecting physical constraints. *In silico *models derived from such reconstructions can then be queried or interrogated through mathematical simulations. Proteomic profiling studies of the mature human erythrocyte have shown more proteins present related to metabolic function than previously thought; however the significance and the causal consequences of these findings have not been explored.

**Results:**

Erythrocyte proteomic data was used to reconstruct the most expansive description of erythrocyte metabolism to date, following extensive manual curation, assessment of the literature, and functional testing. The reconstruction contains 281 enzymes representing functions from glycolysis to cofactor and amino acid metabolism. Such a comprehensive view of erythrocyte metabolism implicates the erythrocyte as a potential biomarker for different diseases as well as a 'cell-based' drug-screening tool. The analysis shows that 94 erythrocyte enzymes are implicated in morbid single nucleotide polymorphisms, representing 142 pathologies. In addition, over 230 FDA-approved and experimental pharmaceuticals have enzymatic targets in the erythrocyte.

**Conclusion:**

The advancement of proteomic technologies and increased generation of high-throughput proteomic data have created the need for a means to analyze these data in a coherent manner. Network reconstructions provide a systematic means to integrate and analyze proteomic data in a biologically meaning manner. Analysis of the red cell proteome has revealed an unexpected level of complexity in the functional capabilities of human erythrocyte metabolism.

## Background

The advancement of high-throughput data generation has ushered a new era of "omic" sciences. Whole-cell measurements can elucidate the genome sequence (genomics) as well as detect mRNA (transcriptomics), proteins (proteomics), and small metabolites (metabolomics) under a specific condition. Though these methods provide a broad coverage in determining cellular activities, little integrated functional analysis has been performed to date.

Genome-scale network reconstructions are a common denominator for computational analysis in systems biology as well as an integrative platform for experimental data analysis [1,2]. There are several applications of reconstructions including: 1) contextualization of high-throughput data, 2) directing hypothesis-driven discovery, and 3) network property discovery [1]. Network reconstruction involves elucidating all the known biochemical transformations in a particular cell or organism and formally organizing them in a biochemically consistent format [3]. Genome sequencing has allowed genome-scale reconstruction of numerous metabolic networks of prokaryotes and eukaryotes [4-6]. In fact, a genome-scale reconstruction of human metabolism has been completed, called Recon 1. Recon 1 is a global human knowledge-base of biochemical transformations in humans that is not cell or tissue specific [7]. More recently, Recon 1 has been adapted to study specific cells and tissues with the help of high-throughput data, including the human brain [8], liver [9,10], kidney [11], and alveolar macrophage [12].

Though many cell and tissue-specific models have been reconstructed from Recon 1, the human erythrocyte has undeservedly received less attention as the cell has been largely assumed to be simple. Historically, red cell metabolic models began with simple glycolytic models [13,14]. In a fifteen year period, the original mathematical model was updated to include the pentose phosphate pathway, Rapoport-Luebering shunt, and adenine nucleotide salvage pathways [15-18]. More recent metabolic models have been built accounting for additional regulatory [19] and metabolic components (e.g. glutathione [20]). However, in the past decade, attempts to obtain comprehensive proteomic coverage of the red cell have demonstrated a much richer complement of metabolism than previously anticipated [21-24]. Modeling the unexpected complexity of erythrocyte metabolism is critical to further understanding the red cell and its interactions with other human cells and tissues.

Thus, we use available proteomics to develop the largest (in terms of biochemical scope) *in silico *model of metabolism of the human red cell to date. Though comprehensive proteomic data provides an overview of red cell proteins, we believe it does not provide a full functional assessment of erythrocyte metabolism. Thus, we have also gathered 50+ years of erythrocyte experimental studies in the form of 60+ peer-reviewed articles and books to manually curate the final model. In order to objectively test physiological functionality, we have put the final model through rigorous simulation.

## Results and Discussion

iAB-RBC-283 is a proteomic based metabolic reconstruction and a biochemical knowledge-base, a functional integration of high-throughput biological data and existing experimentally verified biochemical erythrocyte knowledge that can be queried through simulations and calculations. We first describe the process and characterize the new erythrocyte reconstruction and determine the metabolic functionality. Then, we analyze the results by mapping genetic polymorphisms and drug target information onto the network.

### Proteomic based erythrocyte reconstruction

Proteomic data has been successfully used for reconstructions of *Thermotoga maritima *[25] and the human mitochondria [26] and provides direct evidence of a cell's ability to carry out specific enzymatic reactions. One challenge in the measurement of proteomic data is the depth of coverage, which is still known to be incomplete, even for studies aiming to obtain comprehensive coverage. Another challenge is the possible contamination in the extract preparation with other blood cells [21]. In addition, leftover enzymes from immature erythroid cells are possibly retained in mature red blood cells. With multiple comprehensive proteomic studies carried out in the last decade, the coverage for the red cell has improved significantly but gaps and inaccurate data still plague proteomic studies of the erythrocyte.

**Figure 1:**
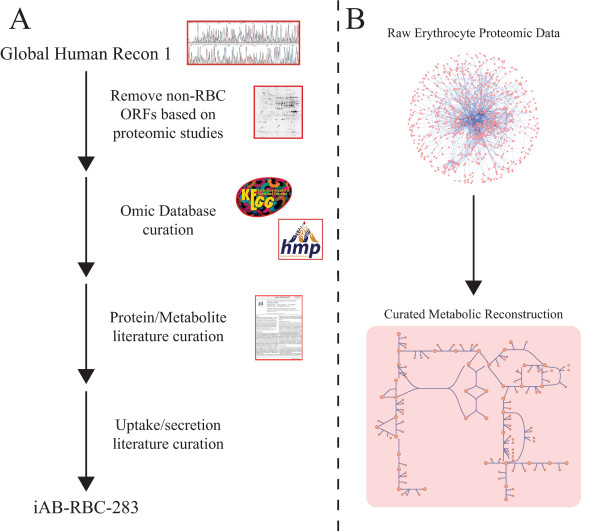
**(A) Workflow for building a comprehensive *in silico *erythrocyte metabolic network**. The three major data types required are: the human genome sequence, high-throughput data (specifically, proteomics for an enucleated cell), and primary literature. The global human metabolic network, Recon 1, was constructed from the human genome sequence and annotation. To build the erythrocyte network, iAB-RBC-283, proteomics was used to remove non-erythrocyte related open reading frames (ORFs) or genes. Detailed curation utilizing protein, metabolite, and transport experimental literature was needed to build a high-quality metabolic reconstruction. (B) Without network reconstruction and rigorous curation, the experimentally generated proteomic data is raw and difficult to interpret. The process detailed in panel A provides a means to build a meaningful knowledge-base of available high-throughput data that can then be probed and tested.

Thus, in this study, we construct a full bottom-up reconstruction of erythrocyte metabolism with rigorous manual curation in which reactions inferred from proteomically detected enzymes were cross-referenced with existing experimental studies and metabolomic data as part of the quality control measures to validate and gap fill metabolic pathways and reactions (Figure 1).

**Figure 2:**
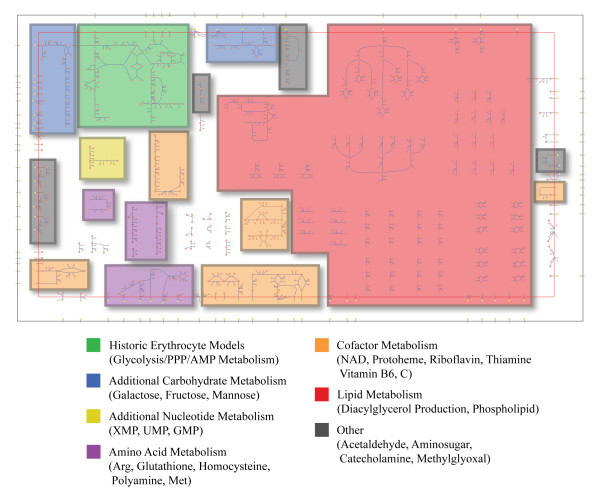
**Topological map of the human erythrocyte metabolic network (iAB-RBC-283)**. Utilizing proteomic data, a much expanded metabolic network was reconstructed accounting for additional carbohydrate and nucleotide metabolism. In addition, the erythrocyte plays roles in amino acid, cofactor, and lipid metabolism. Abbreviations: PPP - pentose phosphate pathway, Arg - arginine, Met - methionine.

The final reconstruction, iAB-RBC-283, contains a metabolic network that is much more expansive than red blood cell models presented to date (Figure 2). The reconstruction contains 292 intracellular reactions, 77 transporters, 267 unique small metabolites, and accounts for 283 genes, suggesting that the erythrocyte has a more varied and expansive metabolic role than previously recognized.

A full bottom-up reconstruction of the human erythrocyte provides a functional interpretation of proteomic data that is biochemically meaningful. Manual curation provides experimental validation of metabolic pathways, as well as gap filling. The data can be rigorously and objectively analyzed through *in silico *simulation.

### Functional assessment of iAB-RBC-283

In order to ascertain the functional capabilities of the expanded erythrocyte reconstruction, iAB-RBC-283 was converted into a mathematical model. The expanded erythrocyte network was topologically and functionally compared to a previous constraint-based model of erythrocyte metabolism [27] (see additional file 1). Predictions made by this model could be recapitulated by iAB-RBC-283. To determine new functionalities of the expanded erythrocyte network, the system is assumed to be at a homeostatic state and qualitative capacity/capability simulations are done to ascertain which reactions and pathways can be potentially active in the *in silico *erythrocyte. Flux variability analysis (FVA) was utilized to determine the functional metabolic pathways of the erythrocyte network (see Materials and Methods). FVA determines the minimum and maximum allowable flux through each metabolic reaction [28]. In short, the FVA method defines the bounding box on network capabilities. Reactions that had a non-zero minimum or maximum flux value were deemed to be functional.

Network level metabolic functional assessment showed that iAB-RBC-283 accounts for additional pathways into glycolysis through galactose, fructose, mannose, glucosamine, and amino sugars (N-Acetylneuraminate). Galactose can also be shuttled to the pentose phosphate pathway through glucuronate interconversions. Citric acid cycle enzymes (fumarase, isocitrate dehydrogenase, and malate dehydrogenase) are present, but we were unable to fully understand their roles as full metabolic pathways were not present. However, fumarate can be shuttled into the model and exported as pyruvate through conversions by fumarase and malic enzyme.

In addition, nucleotide metabolism and salvage pathways have been expanded from previous metabolic models to account for XMP, UMP, GMP, cAMP, and cGMP metabolism. In particular, ATP and GTP can be converted into cAMP and cGMP respectively as adenylate and guanylate cyclases were found to be present in the proteomic data.

The erythrocyte uses amino acids to produce glutathione for redox balancing, converts arginine to polyamines and a byproduct of urea, and utilizes homocysteine for methylation. It has been proposed that Band III is the major methylated protein, particularly for timing cell death [29]. This has also been included in iAB-RBC-283. In addition, polyamine metabolism produces 5-methylthioadenosine which can be salvaged for methionine recycling.

Another major expansion in metabolic capabilities represented in iAB-RBC-283 is lipid metabolism. Though the mature erythrocyte is unable to produce or degrade fatty acids, the cell can uptake fatty acids from the blood plasma to produce and incorporate diacylglyercol into phospholipids for upkeep of its membrane [30]. A pseudo-carnitine shuttle in the cytosol is used to create a buffer of CoA for the cell [31]. All major erythrocyte fatty acids (C16:0, C18:1, C18:2) and phospholipids (phosphatidylcholine, phosphatidylethanolamine, phosphatidylinositol) are explicitly modeled. The phosphatidylinositols can be converted into various forms of myo-inositols, which play an extensive role in cell signaling [32].

Finally, FVA showed that the erythrocyte plays an important role in cofactor metabolism. The reconstruction accounts for uptake, modification, and secretion of multiple cofactors including vitamin B6, vitamin C, riboflavin, thiamine, heme, and NAD. In addition, human erythrocytes play a role in deactivating catecholamines [33], hydrolyzing leukotriene [34], and detoxifying acetaldehyde [35] which were confirmed in the literature.

### Metabolite connectivity

In order to compare the network structure of the *in silico *erythrocyte versus other similar metabolic networks, we calculated the connectivity of each metabolite [36]. Simply, the connectivity is the number of reactions that a metabolite participates in. As a metabolite can be defined as a node in a network structure, the biochemical reactions associated with a particular metabolite are the edges of the network. Metabolite connectivity thus involves determining the number of edges (reactions) connected to every node (metabolite).

**Figure 3:**
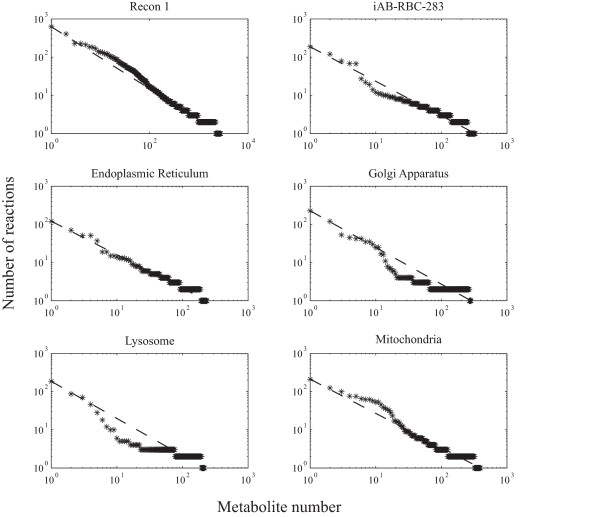
**The metabolite connectivity of iAB-RBC-283, Recon 1, and the similarly sized organelles in Recon 1**. Recon 1 and the mitochondria have high network connectivity, with many data points above the reference lines. The erythrocyte network and other organelles have less metabolic connectivity denoted by data point being on or below the reference lines. This characteristic can be attributed to either 1) an inherently 'fragmented' erythrocyte network, or 2) incomplete proteomic coverage.

We compared the *in silico *erythrocyte to the global human metabolic network, Recon 1, as well as the separate human organelles (Figure 3). A dotted line, linking the minimum and maximum connectivities, is drawn on the distributions as a reference for comparing the distributions. A network with higher connectivity would have many points above the dotted line, while lower connectivity would result in points below the dotted line. Recon 1 has most points above the reference line (Figure 3, first panel). In fact, the metabolite node connectivities for genome-scale reconstructions usually have a distribution similar to Recon 1, highlighting the complexity of these networks [37]. However, the organelles in Recon 1 usually have values below the reference line, due mainly to a higher difficulty to annotate reactions specific to an organelle. The separate organelle metabolic networks are less connected. The exception is the mitochondria (Figure 3, last panel), which is well studied and has a very important and complex metabolic role.

The metabolite connectivity of the erythrocyte network is very similar to the less connected organelles in Recon 1. This similarity is due to either 1) the erythrocyte biology or 2) the lack of complete proteomic profiling. First, as the erythrocyte circulates in blood, it has access to many types of metabolites. As the erythrocyte is relatively simple, it is possible that the reconstructed metabolic network is complete as different types of metabolites do not have to be created and can easily be transported into or out of the cell (e.g. amino and fatty acids). However, the network simplicity of the *in silico *erythrocyte model may also be attributed to the limitations of proteomic techniques. As coverage of proteomic data is typically not as complete as transcriptomics, the reconstructed metabolic network may reflect this limitation. Thus, deeper proteomic profiling could be of great use to further elucidate the role of the erythrocyte in systemic metabolism and the complexity of its own metabolic network.

*In silico *simulations show a greater physiological functionality of erythrocyte metabolism. The additional functionality is not evident from the proteomic data alone. However, metabolite connectivity analysis shows that additional targeted proteomics are of interest. During the manual curation steps we noted that portions of the TCA cycle were detected but not functional. Further studies for cysteine, folate, and phospholipid metabolism are also of interest as some enzymes were detected in the proteomic data but little or no conclusive experimental literature was found.

### The human erythrocyte's potential as a biomarker

Decades of models have described erythrocyte metabolism to include principally glycolysis, the Rapoport-Luebering shunt, the pentose phosphate pathway, and nucleotide salvage pathways. Integration and compilation of proteomic data, however, has surprisingly shown evidence of a much richer metabolic role for the erythrocyte. Erythrocytes make contact with most portions of the body and are one of the most abundant cells (about 2 liters in volume in a typical adult). With such a varied metabolic capacity, the erythrocyte can act as a sink for and source of metabolites throughout the body.

Erythrocytes have been previously studied as potential biomarkers for riboflavin deficiency [38], thiamine deficiency [39], alcoholism [40], diabetes [41], and schizophrenia [42], however comprehensive systems level analyses have not been performed to date.

**Figure 4:**
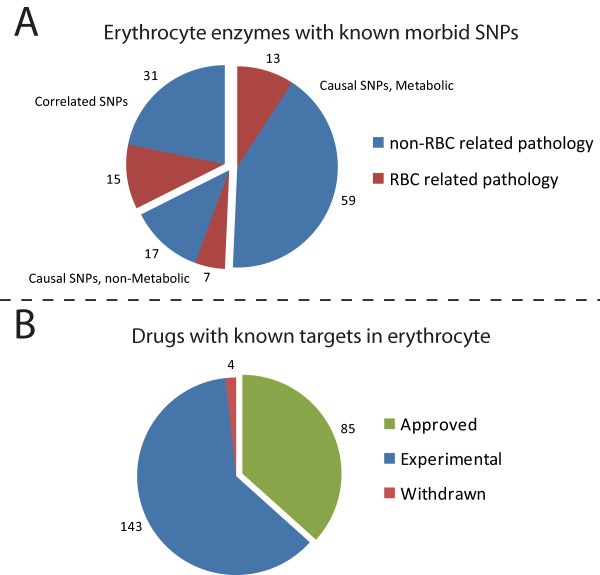
**To test the potential use of erythrocytes as biomarkers, we identified the known morbid SNPs and drug targets of erythrocyte enzymes account for in the reconstructed network**. 142 morbid SNPs were identified with the majority dealing with non-erythrocyte related pathologies (see Table 1). In addition, over 230 FDA-approved, FDA-withdrawn, and experimental drugs have known protein targets in the human erythrocyte.

iAB-RBC-283 explicitly accounts for the genetic basis of the enzymes and transporters that it represents. To determine the capability of the *in silico *erythrocyte as a potential biomarker, we cross-referenced morbid SNPs from the OMIM and nearly 4800 drugs from the DrugBank database with the 281 enzymes accounted for in iAB-RBC-283. 142 morbid SNPs were found in 90 of the erythrocyte enzymes. In addition, 232 FDA-approved, FDA-withdrawn, and experimental drugs have known protein targets in the human erythrocyte (Figure 4).

Only 35 of the 142 morbid SNPs are related to pathologies unique to the erythrocyte, mainly dealing with hemolytic anemia. The majority of the morbid SNPs deal with pathologies that are peripheral to the red blood cell and to the blood system. The remaining non-erythrocyte related pathologies are classified in Table 1 using the Merck Manual [43]. Most of the observed SNPs are causal and simple targeted assays could be used as diagnostic tools.

**Table 1:** Morbid SNPs with non-erythrocyte related pathologies

	Causal SNPs, Metabolic	Causal SNPs, Non-Metabolic	Correlated SNPs
Pediatric Disorders	28	6	4

Endocrine and Metabolic Disorders	10	2	8

Neurologic Disorders	5	2	1

Genitourinary Disorders	4	2	1

Eye Disorders	1	3	2

Musculoskeletal and Connective Tissue Disorders	3	-	1

None	1	-	3

Gastrointestinal Disorders	1	-	2

Hepatic and Billary Disorders	3	-	-

Hematology and Oncology Disorders	-	-	2

Nutritional Disorders	-	-	2

Psychiatric Disorders	-	-	2

Cardiovascular Disorders	1	-	-

Dermatologic Disorders	-	1	-

Ear, Nose, Throat, and Dental Disorders	-	1	-

Immunology, Allergic Disorders	1	-	-

Injuries, Poisoning	1	-	-

Pulmonary Disorders	-	-	1

We also cross-referenced the enzymes in iAB-RBC-283 with the DrugBank's known protein targets of pharmaceuticals. 85 FDA-approved, 4 FDA-withdrawn, and 143 experimental drugs have known targets in the human erythrocyte. These medications target enzymes for the treatment of a wide range of diseases including seizures (topimarate), allergies (flunisolide), cancer (topotecan), HIV (saquinavir), and high cholesterol (gemfibrozil). Due to the availability of erythrocytes from any individual, drugs can be easily screened and optimized *in vitro *for individual patients where the effect of the drug is known to occur in the erythrocyte.

A comprehensive listing of all observed morbid SNPs and drugs are provided in the Supplementary Material (see additional files 2 and 3).

### Utilizing iAB-RBC-283 to develop biomarker studies

**Figure 5:**
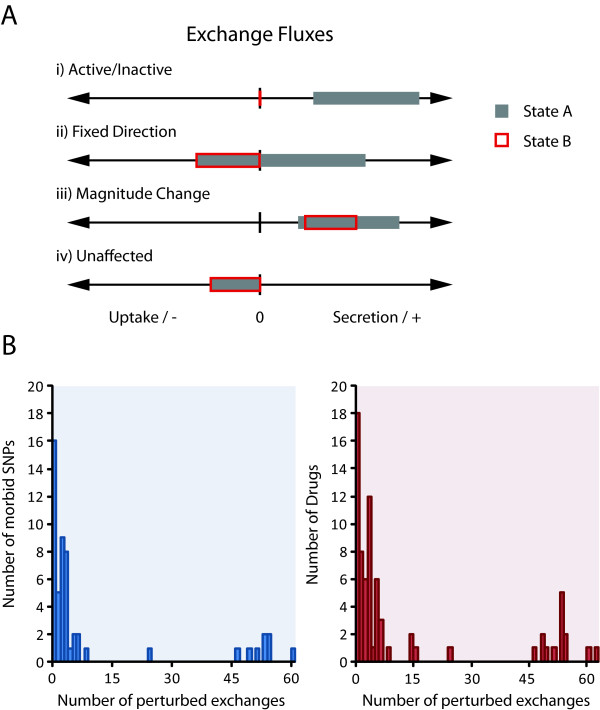
**Flux variability of the exchange reactions can be used to detect metabolic signatures of simulated morbid SNPs and drug treated erythrocytes**. **(A) **Exchange reactions are artificial reactions that allow the mathematical model to uptake and secrete metabolites into the extracellular space. Uptake of a metabolite into the erythrocyte is expressed as a negative value and secretion is expressed as a positive value. There are four major differences that can occur for an exchange reaction in two different states: i) the reaction is either active (non-zero minimum or maximum flux) or inactive (zero minimum and maximum flux), ii) the exchange becomes fixed in one direction (uptake or secretion only), iii) there is a magnitude change in exchange, iv) the reaction is unaffected and is the same for both states. **(B) **We detected 71% and 62% of the morbid SNPs and drugs associated with the erythrocyte. The distribution shows that most perturbed conditions have between one to ten differentially active exchange reactions.

An important application of metabolic reconstructions and the resulting mathematical models is to predict and compare normal and perturbed physiology. We used iAB-RBC-283 to simulate not only normal conditions to study the capacity of erythrocyte function, but also the detected morbid SNPs and drug treated conditions for drugs with known erythrocyte enzyme targets. Flux variability analysis was used to characterize the exchange reactions of the network for determining a metabolic signature in the erythrocyte for the associated perturbed conditions. We compared the minimum and maximum fluxes through each reaction under normal conditions versus all perturbed conditions and determined differential reaction activity (see Methods and Figure 5A). Activated or suppressed flux from *in silico *simulations provides a qualitative understanding into which metabolites and reactions are perturbed, allowing for experimental followup.

We were able to confidently detect *in silico *metabolic flux changes in at least one exchange reaction for 75% of the morbid SNPs and 70% of the drug treated conditions (Figure 5B). On average, there were 12.6 and 9.9 differential activities of exchange reactions for morbid SNPs and drug treated conditions respectively. The average is skewed by some morbid SNPs and drug treated conditions that have over 45 affected exchange reactions, as most differences are detected in between one and ten exchange reactions (Figure 5B).

The morbid SNPs with greater than 45 exchange reactions with differential activity deal mostly with glycolytic and transport enzymes that are known to cause anemias and spherocytosis (see additional file 2 in Supplementary Material). In addition, most of the drug treated conditions with high numbers of differential exchange activity correspond to drugs that have enzyme targets for the same morbid SNPs that are known to cause hemolytic diseases.

An important metabolic enzyme found in erythrocytes is catechol-o-methyltransferase (COMT) used to methylate cathecolamines. A morbid SNP of COMT gene has been implicated in susceptibility to schizophrenia [44]. *In silico *simulations show that the associated morbid SNP erythrocyte has lowered uptake of dopamine and norepinephrine and lowered secretion of the methylated counterparts. Though COMT has not been shown to be causal for schizophrenia, the morbid SNP may have an effect on the phenotype. Qualitative *in silico *simulation of this effect can help focus experimental design on these metabolic pathways in erythrocyte screening.

*In silico *simulations show that the erythrocyte can also be used as a diagnostic for drug treated conditions. For example, topimarate is a drug for treating seizures and migraines and inhibits carbonic anhydrase. Inhibition of this enzyme during simulations showed a drastic change in 54 exchange reaction fluxes pertaining to carbohydrate, cofactor (thiamine, pyridoxine, bilirubin), lipid, and nucleotide metabolism. As individuals react differently to pharmaceuticals and sometimes require different dosages and types of drugs, our analysis shows that the red blood cell can act as a readily available diagnostic for personalizing drug therapies.

To further investigate the diagnostic capability of the red blood cell, we assessed the uniqueness of the metabolic signatures detected. We compared all the metabolite signatures to see if some were shared between different SNPs or drug treatments. In all, 67% of the metabolic signatures are unique with most of the remaining similar to only one other perturbed condition (Table 2).

**Table 2:** Uniqueness of metabolic signatures

	# of Metabolic Signatures	
**# of conditions sharing same met. signature**	**Morbid SNPs**	**Drug Treated**

1 (Unique)	19	19

2	8	3

3	3	1

4	1	1

5	0	1

7	1	0

The *in silico *simulation results provide a method to focus biomarker discovery experiments in the human erythrocyte, as well as interpret global metabolomic profiling. The flux variability shows that a large number of morbid SNPs and drug effects can be detected in the erythrocyte, with most having a unique metabolic signature. The differential activity in exchanges for the perturbed conditions allow for focusing experiments to particular metabolites, exchanges, and associated pathways, allowing development of targeted assays. In addition, global metabolomic profiling of perturbed conditions can be interpreted using the calculated metabolic signatures and the erythrocyte reconstruction. A full listing of all detected morbid SNPs and drug treated conditions, as well as the corresponding exchange reactions with differential activity and fluxes is provided in the Supplementary Material (see additional files 2 and 3).

## Conclusion

The mature, enucleated erythrocyte is the best studied human cell for metabolism due to its relative simplicity and availability. Still, the view of its metabolism is rather limited. The advances in high-throughput proteomics of the erythrocyte has enabled construction of a comprehensive *in silico *red blood cell metabolic reconstruction, iAB-RBC-283. Proteomic data alone is not adequate for generating an accurate, complete, and functional model. The reconstruction was rigorously curated and validated by experimental literature sources as proteomic samples are known to be incomplete, contaminated with other types of blood cells and inactive enzymes are passed down the erythrocyte differentiation lineage. Thus, iAB-RBC-283 is a knowledge-base of integrated high-throughput and biological data, which can also be queried through simulations.

Functional testing showed that the new reconstruction takes into account historically neglected areas of carbohydrate, amino acid, cofactor, and lipid metabolism. Traditionally, the erythrocyte is known for its role in oxygen delivery, but the varied metabolism the cell exhibits points towards a much more expanded metabolic role as the cell can act as a sink or source of metabolites, through interactions with all organs and tissues in the body.

Metabolite connectivity analysis showed that the erythrocyte metabolic network is relatively simple and is similar to human organelles in network structure. This could be due either to shortcomings of the high-throughput data or the relatively simple metabolism of red cells. From our manual curation steps, targeted proteomic studies would be useful for a few metabolic pathways: including TCA cycle, cysteine, folate, and phospholipid metabolism.

A metabolically rich and readily available erythrocyte can be useful for clinical biomarkers. To determine potential uses, we cross-referenced the enzymes in iAB-RBC-283 with known morbid SNPs and enzymes that are reported drug targets in DrugBank. There are 142 morbid SNPs detectable in erythrocyte enzymes with the majority dealing with non-erythrocyte related pathologies. In addition, over 230 pharmaceuticals in the DrugBank have known protein targets in the human erythrocyte.

Utilizing iAB-RBC-283, we qualitatively detected metabolic signatures for the majority of *in silico *perturbed conditions pertaining to the morbid SNPs and drugs from DrugBank. The affected exchange reactions, metabolites, and associated pathways can be used to focus experiments for biomarker discovery as well as interpret global metabolomic profiles.

Taken together, with available proteomic data, a comprehensive constraint-based model of erythrocyte metabolism was developed. Genome-scale metabolic reconstructions have been shown to be an important tool for integrating and analyzing high-throughput data for biological insight [8,12,45,46]. In this study, we show that the comprehensive metabolic network of the erythrocyte plays an unanticipated, varied metabolic role in human physiology and thus has much potential as a biomarker with clinical applications. As erythrocytes are readily available, the proteomics and metabolomics of normal and pathological states of individuals can be easily obtained and used for identifying biomarkers in a systems context.

## Methods

### Reconstructing the comprehensive erythrocyte network

Metabolic network reconstruction has matured into a methodological, systematic process with quality control and quality assurance steps that can be carried out according to standardized detailed protocols [3]. The sequencing of the human genome enabled a comprehensive, global reconstruction for all human cell types to be carried out, with the caveat that in order to implement any context, condition, or cell specific analysis, one would need specific data for the particular human cell or tissue of interest.

Metabolic reconstructions contain all known metabolic reactions of a particular system. The reactions are charge and elementally balanced, with gene-protein-reaction (GPR) annotations. GPRs mechanistically connect the genome sequence with the proteome and the enzymatic reactions. GPRs provide a platform for integration of high-throughput data to model specific conditions. In this study, proteomic data was integrated with Recon 1 to build a comprehensive erythrocyte network.

iAB-RBC-283 was reconstructed in the following manner. Proteomic data for erythrocytes from multiple sources [21-24] were consolidated and cross-referenced with Recon 1. The proteomic data was provided in the IPI format and was converted to Entrez Gene Ids. The Entrez Ids were linked with the Recon 1 transcripts, including alternatively spliced variants, and used to generate a list of potential erythrocyte reactions. Reactions and pathways from Recon 1 that were present in the proteomic data were used to build an automated draft reconstruction. However, blood cell contamination and remnant enzymes from immature erythroid cells decrease the accuracy of algorithmically derived models based on high-throughput data. In order to build the most complete and accurate final model, the draft reconstruction was rigorously and iteratively manually curated. In brief, manual curation involves (1) resolving all metabolite, reaction, and enzyme promiscuity, (2) exploring existing experimental literature to determine whether or not detected enzymes in the proteomics data are correct, (3) determining and filling gaps in the proteomic data through topological gap analysis and flux-based functional tests. Steps 2 and 3 are an iterative process where new biochemical data increases the scope of the network, requiring additional gap and functional analysis as well as additional literature mining.

Thus, possible remnant enzymes, such as glycogen synthase and aspartate aminotransferase, were properly removed when experimental validation was not available. The manual curation process yielded 60+ peer reviewed articles and books representing over 50 years of erythrocyte research. In addition, erythrocyte literature sources from the Human Metabolome Database [47] were used for validating existence of unique metabolites. A full listing of literature sources for each reaction, enzyme, and metabolite is provided in the Supplementary Material (see additional file 4, iAB-RBC-283 in XLS format).

Fatty acid chains were not represented as generic R-groups as in Recon 1, but instead the three most common (by percent mass composition) fatty acids, palmitic, linolenic, and linoleic (C16:0, C18:1, C18:2, respectively) were used [48]. The final reconstruction is termed iAB-RBC-283 for i (*in silico*), AB (the primary author's initials), RBC (red blood cell), 283 (number of open reading frames accounted for in the network). iAB-RBC-283 consists of all the known metabolites, reactions, thermodynamic directionality, and genetic information that the detected erythrocyte metabolic enzymes catalyze. The final reconstruction is provided in both an XLS and SBML format in the Supplementary Material (additional files 4 and 5) and can also be found at the BioModels Database (id: MODEL1106080000).

### Constraint-based modeling and functional testing

The network reconstruction can be represented as a stoichiometric matrix, **S**, that is formed from the stoichiometric coefficients of the biochemical transformations. Each column of the matrix represents a particular elementally and charge balanced reaction in the network, while each row corresponds to a particular metabolite [37]. Thus, the stoichiometric matrix converts the individual fluxes into network based time derivatives of the concentrations (Equation 1).(1)

As each reaction is comprised of only a few metabolites, but there are many metabolites in a network, each flux vector is quite sparse. The stoichiometric matrix is sparse, with 1.3% non-zero elements, and has dimensions (**m **× **n**); where **m **is the number of compounds and **n **is the number of reactions. In the case of iAB-RBC-283, **m **is 342 and **n **is 469.

As *in vivo *kinetic parameters are difficult to obtain, the system is assumed to be at a homeostatic state (Equation 2),(2)

allowing for simulation without kinetic parameters. The network fluxes (**v**) are bounded by thermodynamic constraints that limit the directionality of irreversible catalytic mechanisms (lb = 0 for irreversible reactions) as well as known v_max_'s.(3)

Thus, the network is studied under mass conservation and thermodynamic constraints. In addition, constraints are placed on fluxes that exchange metabolites with the surrounding system (the blood plasma in this case), based on existing literature of metabolite transport in the human erythrocyte (see additional file 6 in Supplementary Material). These reactions are called exchange reactions and control the flow of metabolites into and out of the *in silico *cell.

Flux balance analysis (FBA) is a well-established optimization procedure [37] used to determine the maximum possible flux through a particular reaction in the network based on the constraints on the network (Equations 2 and 3) without the need for kinetic parameters. A primer for using FBA and related tools is detailed by Orth et al. [49]. Publicly available software packages exist [50]

In this work, a variant of FBA, called flux variability analysis (FVA) [28], is used. FVA iteratively calculates both the maximum and minimum allowable flux through every reaction in the network. Reactions with a calculated non-zero maximum or minimum have the potential to be active and have a potential physiological function. Thus, we use FVA to determine the capability/capacity of the network reactions to determine metabolic functionality. For a reaction to have a non-zero flux, the reaction must be linked to other metabolic reactions and pathways and plays a functional role in the system. Thus, potentially active reactions are deemed as functional. After determining which reactions were functional, the reaction list was perused to determine pathway and subsystem functionality in the network.

### Calculating metabolite connectivity

The stoichiometric matrices of the reconstructed erythrocyte network, Recon 1, and its constituent organelles were used to calculate metabolite connectivities [36] of every species in each network. The number of reactions each metabolite participates in was summed. For the organelle calculations, only the metabolites corresponding to the particular organelle of Recon 1 were considered. The metabolite connectivity of each organelle as well as Recon 1 and iAB-RBC-283 was ranked order from greatest to least connected to form a discrete distribution (Figure 3).

### Analyzing iAB-RBC-283 as a functional biomarker

The Morbid Map from the Online Mendelian Inheritance in Man (OMIM) [51] and the DrugBank [52] were downloaded from their respective databases (accession date: 27/09/10). The enzyme names in iAB-RBC-283 were cross-referenced against the database entries to determine morbid SNPs in erythrocyte proteins and drugs with protein targets in the erythrocyte. The morbid SNPs that did not have sole pathological effects in the erythrocyte were classified using the Merck Manual [43].

Just as FVA can be used to assess the function of a network under a particular set of constraints, it can also be used to assess the changes in function and thus has applications for characterizing disease states [53] and identifying biomarkers [54]. When simulating a morbid SNP or a drug inhibited enzyme, the lower and upper bound constraints on the affected reaction is set to zero as per Shlomi et al. FVA is then used to characterize the exchange reactions under morbid SNP or drug treated conditions (Figure 5A) and then compared to the normal state. A reaction was considered to be confidently altered if the change in the minimum or maximum flux was 40% of the total flux span. The flux span is defined as the absolute difference between the original (unperturbed) maximum and minimum fluxes. Potential thresholds from 5 - 60% were tested. Thresholds in the 15 - 40% range were very consistent while thresholds above 40% had a dropoff (see additional file 7).

## Authors' contributions

AB reconstructed the network, performed the analyses, and drafted the manuscript. NJ and BOP conceived the study and revised the manuscript. All authors approved the content of the final manuscript.

## Supplementary Material

Additional file 1 **Comparison of iAB-RBC-283 and previous constraint-based model of human erythrocyte**. iAB-RBC-283 was compared with the previous constraint-based model of the human erythrocyte. Topological analysis showed that iAB-RBC-283 is a much more expansive network that better describes human erythrocyte metabolism. In addition, we recapitulated the randomized sampling results of the previous network and showed that the new erythrocyte model is more accurate, capturing all the correct predictions of the previous model but also correcting its inaccurate predictions. Click here for file

Additional file 2 **Detected SNPs and FVA results for SNP perturbations**. Tables containing the OMIM SNPs of enzymes that are in iAB-RBC-283 with known pathologies, symptoms, metabolic subsystem, and classification. In addition, exchange reactions determined by FVA to be different in SNP perturbations are provided. Click here for file

Additional file 3 **Detected drug targets and FVA results for drug effect perturbations**. Tables containing the known drugs, from DrugBank, that have targets in enzymes that are in iAB-RBC-283. In addition, information is provided on the drug including name, description, and classification. The exchange reactions determined by FVA to be different in drug perturbations are also provided. Click here for file

Additional file 4 **iAB-RBC-283 Reconstruction (table format)**. The reconstruction is provided in a table format with reactions, metabolites, and gene-protein-reaction associations. In addition, information is provided on whether the reaction was detected in the proteomic or metabolomic data and citations are provided for reactions with existing experimental evidence, implicating the reactions presence in the human erythrocyte. Click here for file

Additional file 5 **iAB-RBC-283 Reconstruction (SBML format)**. The reconstruction is provided in the standardized SBML format. The XML file can be loaded in to COBRA toolbox to perform *in silico *simulations. A copy of the file is also available at the BioModels Database (id: MODEL1106080000). Click here for file

Additional file 6 **Citations for exchanges in the human erythrocyte**. Table containing citations used to determine exchange rates of metabolites into the human erythrocyte. Click here for file

Additional file 7 **Parameter sensitivity of threshold for FVA simulations**. Figure showing the average number of FVA-detected exchange reactions for each perturbation and different thresholds. Thresholds were tested from 5-60% at intervals of 5%. The average detected reactions were quite stable from 15-40% for both the SNP and drug perturbations. A final 40% threshold was used in the study. Click here for file
