# Supplementary material for: iAB-RBC-283: A proteomically derived knowledge-base of erythrocyte metabolism that can be used to simulate its physiological and patho-physiological states
Source: BMC Syst Biol. 2011 Jul 12;5:110. doi: 10.1186/1752-0509-5-110 (PMC3158119; doi:10.1186/1752-0509-5-110)
Supplement: Additional file 1 — Comparison of iAB-RBC-283 and previous constraint-based model of human erythrocyte. iAB-RBC-283 was compared with the previous constraint-based model of the human erythrocyte. Topological analysis showed that iAB-RBC-283 is a much more expansive network that better describes human erythrocyte metabolism. In addition, we recapitulated the randomized sampling results of the previous network and showed that the new erythrocyte model is more accurate, capturing all the correct predictions of the previous model but also correcting its inaccurate predictions. [file 1752-0509-5-110-S1.DOC]

*Comparison of Metabolic Erythrocyte Models*

iAB-RBC-283 represents an expanded constraint-based model of human erythrocyte metabolism compared to the constraint-based erythrocyte model used in previous works (Table 1). Though there are numerous kinetic models of erythrocyte metabolism (see Background in main text), we focused on the previous constraint-based network in order for better comparison. The previous model accounts for glycolysis, the Rapoport-Leubering shunt, pentose phosphate pathway, and AMP metabolism. iAB-RBC-283 incorporates the functionality of the previous model but also captures additional carbohydrate and nucleotide metabolism. The network also incorporates portions of human amino acid, cofactor, and lipid metabolism not described in any previous erythrocyte model (see Results in main text). Even when the exchange constraints of iAB-RBC-283 are set to those of the older erythrocyte model, the network is still much larger (Table 1).

Price et al. used the previous erythrocyte network for simulating enzymopathies through Monte Carlo sampling and correlating steady-state solution flux space size with hemolytic anemia . We used iAB-RBC-283 with the exchange constraints of the older model to recapitulate the previous results to show the new model’s validity and increased accuracy. We separately constrained hexokinase (HK), phosphofructokinase (PFK), triose phosphate isomerase (TPI), aldolase (ALD), pyruvate kinase (PK), enolase (EN), phosphoglucoisomerase (PGK), phosphoglycerate kinase (PGK), diphosphoglycerate phosphatase (DPGase), diphosphoglyceromutase (DPGM), and lactate dehydrogenase (LDH) and sampled the space using a similar approach as Bordbar et al. . The size of the solution space was calculated by integrating the area under the histogram curves for all the reactions. As expected, the raw percentages were different between the iAB-RBC-283 results and those obtained by Price et al. due to 1) a different sampling approach was used, 2) a different method was used for calculating flux space volume, and 3) the constrained iAB-RBC-283 has many more dimensions and degrees of freedom than the old constraint-based erythrocyte model. There are quantitative differences for the two models, but the qualitative results and the interpretation remain the same and in the case of LDH, the new model is more accurate. The difference of percentages between the hemolytic anemia causal enzymes (HEX/PFK/TPI/ALD/PK/EN/PGI/PGK) and non-causal enzymes (DPGM/DPGase) are still apparent. In addition, the previous model was inaccurate as it predicted LDH to be constraining but an enzymopathy of lactate dehydrogenase does not cause hemolytic anemia. Simulations with iAB-RBC-283 for perturbed LDH resulted in an unconstrained network, showing an increased accuracy of the expanded model. Thus, iAB-RBC-283 is more accurate and robust than the previous constraint-based erythrocyte model (Table 2). iAB-RBC-283 is a proteomic based reconstruction of the erythrocyte that has many more metabolic functionalities than previously anticipated and modeled. In addition, the network has the ability to capture the similar and more accurate functional characteristics of previous models.

Supplemental Table 1: Network size characteristics of iAB-RBC-283 and the previous constraint-based erythrocyte model

|  | Old RBC | iAB-RBC-283 | iAB-RBC-283 (constrained) |
| --- | --- | --- | --- |
| Genes | - | 283 | 152 |
| Intracellular Reactions | 32 | 292 | 197 |
| Exchange Reactions | 19 | 77 | 13 |
| Metabolites | 39 | 267 | 153 |

Supplemental Table 2: Size of flux solution spaces in enzymopathic cases versus ‘normal’ (i.e. no enzympathy).

| Deficient Enzyme | iAB-RBC-283 | Price et al. | Hemolytic Anemia |
| --- | --- | --- | --- |
| HK | 19.67% | 0.02% | Yes |
| PFK | 23.01% | 0.21% | Variable |
| TPI | 29.39% | 0.21% | Yes |
| ALD | 39.72% | 0.21% | Yes |
| PK | 37.04% | 0.83% | Yes |
| EN | 38.77% | 0.84% | Yes |
| PGI | 27.82% | 1.0% | Yes |
| PGK | 23.66% | 2.2% | Usually |
| DPGase | 100% | 29% | No |
| DPGM | 97.03% | 47% | No |
| LDH | 100% | 0.56% | No |

References:

1. Wiback, S.J. and Palsson, B.O., *Extreme pathway analysis of human red blood cell metabolism.* Biophysical Journal, 2002. **83**(2): p. 808-818.

2. Price, N.D., Schellenberger, J., and Palsson, B.O., *Uniform Sampling of Steady State Flux Spaces: Means to Design Experiments and to Interpret Enzymopathies.* Biophysical Journal, 2004. **87**(4): p. 2172-86.

3. Bordbar, A., Lewis, N.E., Schellenberger, J., Palsson, B.O., and Jamshidi, N., *Insight into human alveolar macrophage and M. tuberculosis interactions via metabolic reconstructions.* Mol Syst Biol, 2010. **6**: p. 422.

4. Tanaka, K.R. and Zerez, C.R., *Red cell enzymopathies of the glycolytic pathway.* Seminars in Hematology, 1990. **27**(2): p. 165-185.
