# Supplementary figures and images for: iAB-RBC-283: A proteomically derived knowledge-base of erythrocyte metabolism that can be used to simulate its physiological and patho-physiological states
Source: BMC Syst Biol. 2011 Jul 12;5:110. doi: 10.1186/1752-0509-5-110 (PMC3158119; doi:10.1186/1752-0509-5-110)

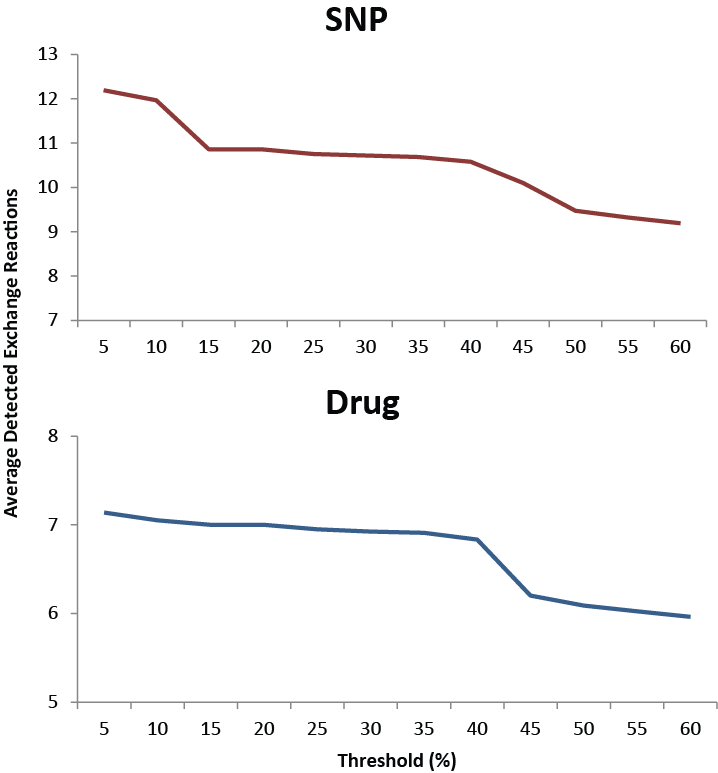

Supplement: Additional file 7 — Parameter sensitivity of threshold for FVA simulations. Figure showing the average number of FVA-detected exchange reactions for each perturbation and different thresholds. Thresholds were tested from 5-60% at intervals of 5%. The average detected reactions were quite stable from 15-40% for both the SNP and drug perturbations. A final 40% threshold was used in the study. [file 1752-0509-5-110-S7.PNG]
